# Three-Dimensional Printed Model and Virtual Reconstruction: An Extra Tool for Pediatric Solid Tumors Surgery

**DOI:** 10.1055/s-0038-1672165

**Published:** 2018-10-18

**Authors:** Ángela Sánchez-Sánchez, Óscar Girón-Vallejo, Ramón Ruiz-Pruneda, Maria Fernandez-Ibieta, Darío García-Calderon, Vanesa Villamil, María Cristina Giménez-Aleixandre, Carlos Andrés Montoya-Rangel, Juan Pedro Hernández Bermejo

**Affiliations:** 1 Department of Pediatric Surgery, Clinical University Hospital Virgen de la Arrixaca, Murcia, Spain; 2 Cathedra of Multidisciplinary Oncology, UCAM, Murcia, Spain

**Keywords:** virtual reconstruction, 3D printing, stereolithography, oncology, pediatrics

## Abstract

**Introduction** Three-dimensional (3D) technology is increasingly applied for planning challenging surgical interventions. We report our experience using 3D printing and virtual reconstruction for surgical planning of complex tumor resections in children.

**Methods** Data were obtained from preoperative magnetic resonance. imaging analysis and 3D virtual recreations were performed using specialized computer software. 3D real-scale geometry models, including tumor, adjacent organs, and relevant vascularization, were printed in colorimetric scale and different materials for optimal structures discrimination.

**Results** Four complex cases were selected. The first case was a bilateral Wilms tumor. The volumetric reconstruction proved the presence of enough healthy renal tissue, allowing bilateral nephron-sparing surgery. In the second case, reconstruction contributed to the location of pulmonary metastases. The third case was an abdominal neuroblastoma stage L2. The 3D model was of high value for planning and as a reference during the intervention. The last case is a cervico-thoracic neuroblastoma with an anatomopathological diagnosis of ganglioneuroma, located at the cervico-mediastinal juncture, in close relationship with the cervical vessels.

**Conclusions** 3D reconstruction and the full-scale printing models are a useful tool in cases of complex tumor resections as they contribute to a better understanding of the relationships between the tumor and adjacent organs, helping to anticipate certain surgical complications. They also provide additional information to conventional imaging tests, being able to influence therapeutic decisions and facilitate the understanding by the family, improving doctor–patient communication.

## Introduction

Surgical resection is one of the cornerstones in the multidisciplinary treatment of pediatric solid tumors. Preoperative tumor evaluation and surgical strategy planning are commonly based on conventional imaging techniques: ultrasound, nuclear magnetic resonance imaging (MRI), and computed tomography (CT). These tests give us images in two dimensions. Virtual three-dimensional (3D) reconstruction using specialized software provides a better understanding of the spatial relationships between the tumor and adjacent organs and allows exploring in an interactive form the surgical anatomy preoperatively. They also allow a fast and simple representation of high-definition 3D images, using files digital image and communication in medicine (DICOM) from CT or MRI. 1 2 3

This helps to evaluate more accurately the conditions under which the operation will be performed and to anticipate possible intraoperative complications.

3D printing models, also called additive manufacturing models, are the result of the transformation of 3D digital files into tangible models. 4 5 6

This technology began in the 80s when Charles W. Hull designed the first stereolithography machine. Although there are different types of 3D printer, all are based on the fusion or deposition of materials layer by layer until the production of the desired structure. 6 7 8

Recently, numerous studies have been published documenting the usefulness of 3D technology in medicine both from the educational point of view and for planning and surgical training. 5 6 7 8 9

In this article, we present four cases of pediatric patients affected from solid tumor lesions in which 3D reconstruction and printing technology was used in surgical planning, as they were cases in which the surgery was technically complex and or the complete resection presented an important forecast implication.

## Materials and Methods

We conduct a case series study. Data about complex oncological cases in which the 3D reconstruction and printing technology was implemented were collected from January 2016 to January 2018.

The data for the 3D reconstruction were obtained in the DICOM format from the preoperative tests performed on the patients, MRI in three cases and CT in one, without any additional imaging tests. MRI was performed by 1.5-T scanner obtaining coronal and axial sections. Fast Spin Echo T2-fat-suppressed sequences were selected. The slice thickness was 4 to 5 mm. CT images were acquired by high-resolution multiplanar scan of 0.5-mm slice thickness.

Subsequently, the specialized team of engineers of Cella Medical Solutions (Murcia, Spain) performed the processing of two-dimensional images using previously validated medical image treatment advanced algorithms. Initially, a noise reduction algorithm (anisotropic diffusion filter) was applied, followed by segmentation algorithms of the anatomical structures of interest and creation of the 3D image of each structure. These images were exported in sterolitographic file (stl). Finally, the post-processing of the virtual reconstruction was performed.

**Fig. 1:**
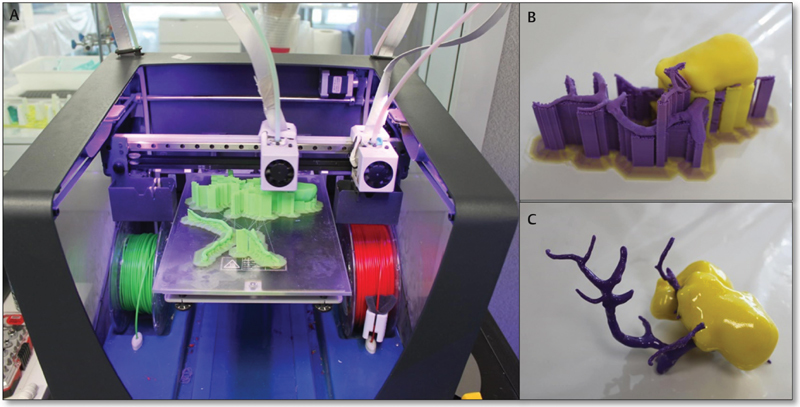
Different steps of 3D printing are shown. ( **A** ) FDM printer; ( **B** ) printed structure still with support material. ( **C** ) Result after post-processing the piece. FDM, fused deposit modeling; 3D, three-dimensional.

After virtual reconstruction was completed, the models were printed by fused deposit modeling (FDM) and injection printing 3D technology (ROVA3D, BCN3D, ORD Solutions, BCN technologies©, and FORM 3D©) ( Fig. 1 ). Different plastic derived materials were used such as polylactic acid, acrylonitrile butadiene styrene (ABS), polyvinyl, polyurethane, and polystyrene. Once the printing was finished, post-processing procedures were performed by removing support structures, clearing coating, and dyeing structures when necessary for getting the most optimal final model.

The printed models were made on real scale, including both the tumor and adjacent organs considered of interest. Special importance was given to the reconstruction of the vascularization involved.

## Results

Between January 2016 and January 2018, data about four cases of patients affected by complex solid tumors were collected, due to both, the location and the relationships of the tumor, as the therapeutic implications of the surgical resection.

**Fig. 2:**
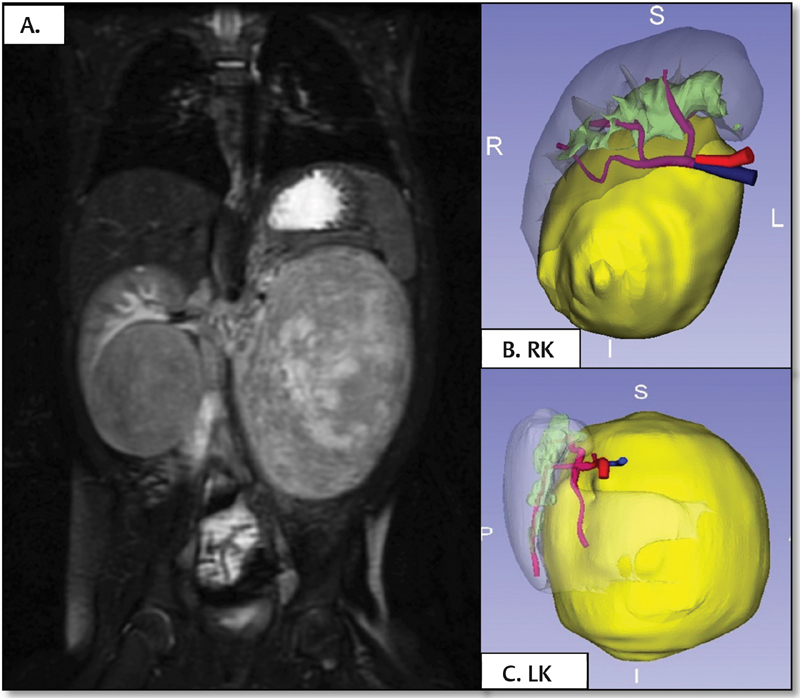
Case 1. ( **A** ) Coronal MRI section showing both kidneys affected by Wilms tumor, confirming the large size of the left tumor mass. ( **B** and **C** ) Volumetric reconstructions of both tumors. Healthy renal parenchyma susceptible to nephron sparing surgery was proved. LK, left kidney; MRI, magnetic resonance imaging; RK, right kidney.

**Fig. 3:**
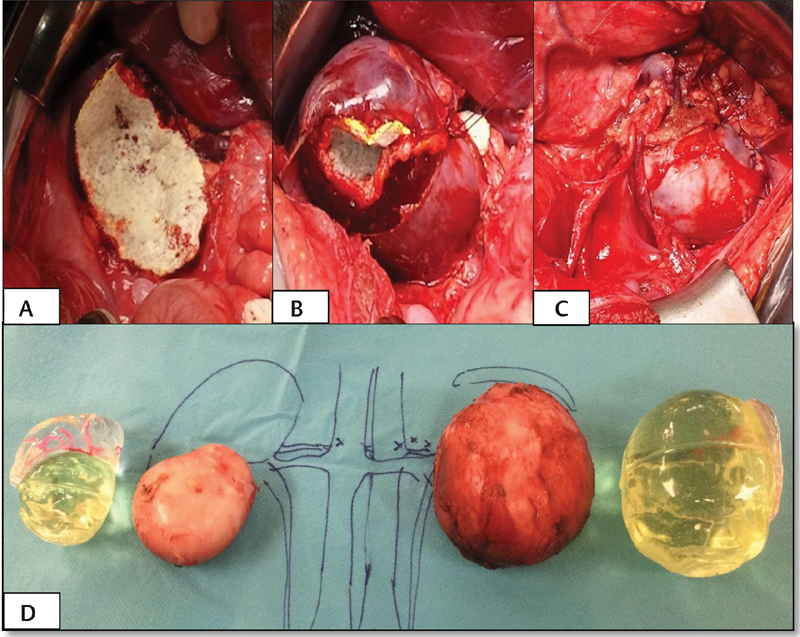
Case 1. The images ( **A** – **C** ) show different moments of the surgical intervention. After bilateral nephron-sparing surgery, coagulation was performed with adhesive matrix and plication of the renal parenchyma. In the image ( **D** ), the resected specimens are shown together with their corresponding three-dimensional models, objectifying the great similarity between both.

**Case 1** : A 7-month-old infant was diagnosed with bilateral Wilms tumor. Neoadjuvant chemotherapy treatment was initiated according to the protocol of the International Society of Pediatric Oncology (SIOP) with little response to chemotherapy. In the preoperative MRI, a large tumor dependent on the left kidney with limited renal parenchyma was observed. Right renal tumor presented smaller size with largely conserved parenchyma. Initially, left radical nephrectomy and right nephron-sparing surgery were planned. The volumetric reconstruction ( Fig. 2 ) of both the tumor and the healthy renal tissue proved that the left kidney parenchyma free of nephroblastoma was greater than the bidimensional imaging tests showed, considering feasible to perform bilateral nephron-sparing surgery. Using 3D printing technology, 1:1 scale models were obtained providing information on the volume of both tumors in relation to the size of the kidneys and the small size of the renal vascularization. The 3D physical models were consulted during the procedure due to doubts regarding the vascularization of both tumors ( Fig. 3 ). Surgical intervention was accomplished without incidents, but during the postoperative period, the patient presented thrombosis of the right renal artery that was tried to be solved by endovascular treatment, in spite of which atrophy of the right kidney developed. Currently, the patient is in remission of his disease, he maintains normal renal function values although he has hypertension controlled with amlodipine.

**Fig. 4:**
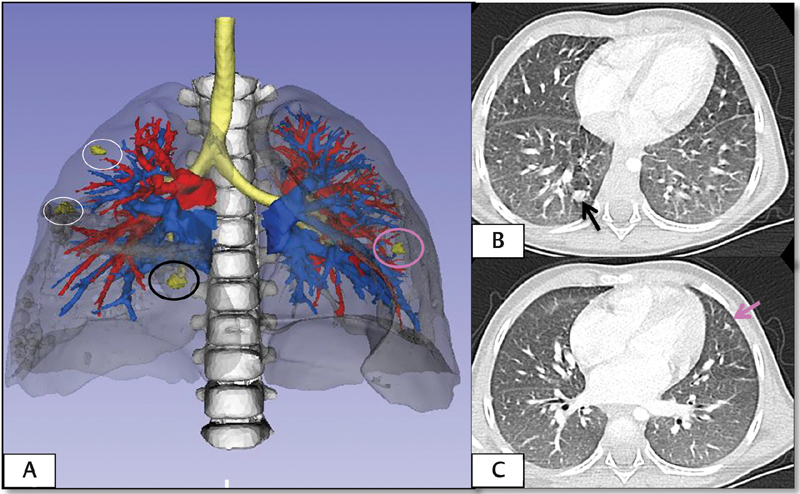
Case 2. ( **A** ) Three-dimensional reconstruction performed from the preoperative CT. There are three metastases in the right lung and one in the left one. ( **B** and **C** ) Cross-sections of the thoracic CT. Lesions marked by arrow correspond to those marked with the same color in the virtual reconstruction. CT, computed tomography.

**Case 2** : A 3-year-old girl was diagnosed with multiple bilateral pulmonary metastases secondary to unilateral, stage III, intermediate risk Wilms tumor. After receiving chemotherapy for metastatic recurrence according to the SIOP protocol, 3 four residual lesions were verified in the control CT; three of them were located in the right lung (two in the right upper lobe and one in the posterior segment of the right lower lobe) and one in the upper lobe of the left lung. Fig. 4 shows the reconstruction of the metastatic lesions and the comparison with their location in the CT scan. The 3D reconstruction allowed the 360-grade vision of the metastases within the pulmonary parenchyma, providing relevant information for a more precise location of the lesions.

**Fig. 5:**
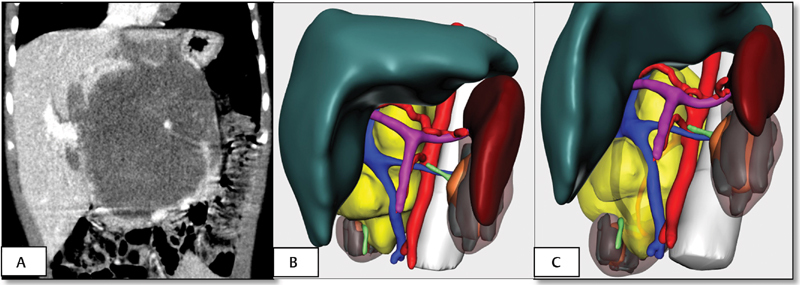
Case 3. ( **A** ) MRI showing a large abdominal mass that moves the liver upward. ( **B** ) Virtual reconstruction of the tumor (yellow), liver (green), both kidneys (gray), and spleen (brown). The involved vasculature was also reconstructed (aorta in red, vena cava in blue, and portal vein in purple). ( **C** ) Modified virtual reconstruction, observing the tumor of semitransparent consistency, which allows the visualization of the renal artery and vein within the tumor. The kidney is displaced inferiorly with loss of its morphology due to the mass effect. MRI, magnetic resonance imaging.

**Fig. 6:**
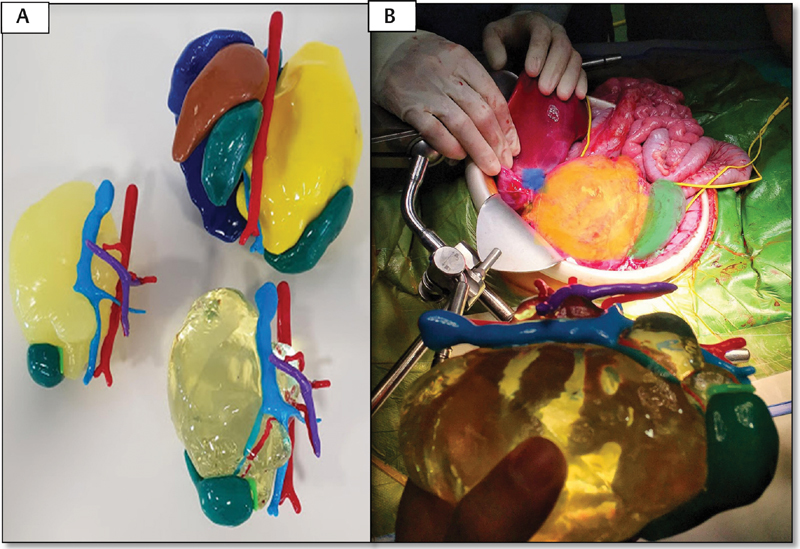
Case 3. ( **A** ) Different printed models representing the abdominal neuroblastoma. The superior model includes tumor (yellow), aorta (red) and vena cava (blue) with its respective branches, liver (blue), both kidneys (green), and spleen (brown). The different structures are assembled by a magnetization system. The other two models represent the tumor and the relevant vascularity in different textures. ( **B** ) The model was consulted several times during the surgery. In the image, the correspondence between the model and the surgical anatomy is observed.

**Case 3** : An infant of 13 months was diagnosed at 7 months of right abdominal neuroblastoma. In MRI, an extensive mass was observed that encompassed the regional aorta, the exit of the celiac trunk, the mesenteric artery, and the renal arteries, exerting mass effect on the inferior vena cava and displacing both the liver and the right kidney. Percutaneous puncture was performed with an anatomopathological diagnosis of poorly differentiated neuroblastoma, classified as Group “D” (low) of the International Neuroblastoma Risk Group (INRG) classification (stage L2, age less than 18 months, MYCN not amplified and 11q not deleted). The abdominal mass was considered unresectable, and an expectant attitude was adopted initially. During follow-up, hypertension and increased compression of the inferior vena cava were found. These symptoms were considered life-threatening; therefore, chemotherapy was initiated according to the European Low and Intermediate Risk Neuroblastoma protocol. The non-response to chemotherapy and the persistence of symptoms led to proposing surgery. Fig. 5 shows an axial section of the MRI showing the large size of the neuroblastoma and the correspondence with the virtual reconstruction in which it is clearly visualized how the tumor includes the superior mesenteric artery and the renal artery, making possible to know the disfigured anatomy by the large abdominal mass. Three different physical models were printed, two models that recreated the tumor, the right kidney and the relevant vessels (vena cava and portal vein, aorta, celiac trunk, and superior mesenteric artery), one of them with the tumor printed in transparent polyurethane for visualization of the vasculature embraced within the tumor. In the other model, liver, spleen and left kidney were also included. In addition to planning the surgical approach, these models were also consulted several times as a guide during the intervention ( Fig. 6 ). Surgery was performed in one of the spanish national reference centers for neuroblastoma obtaining complete resection of the tumor without incidents during the intervention.

**Fig. 7:**
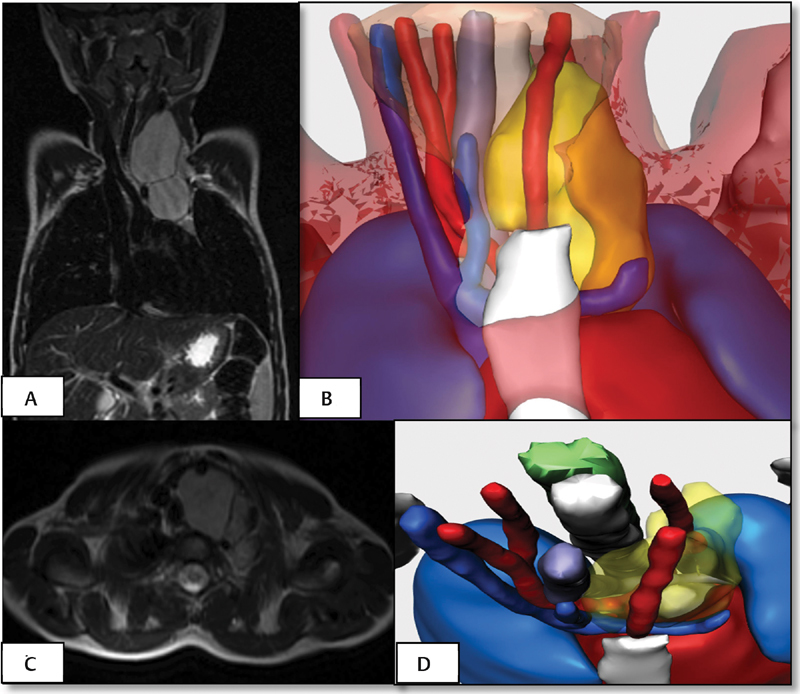
Case 4. Virtual reconstruction of cervicothoracic paravertebral ganglioneuroma and comparison with images obtained by MRI (A-B coronal view, C-D transverse view). The tumor displaces the left carotid artery, the internal jugular vein, and the airway. The vertebral artery is encased by the tumor. MRI, magnetic resonance imaging.

**Case 4** : A 5-year-old girl was suffering from left paravertebral neuroblastoma. She was diagnosed prenatally of pleural effusion, and admission to the neonatal intensive care unit (ICU) was needed at birth due to respiratory insufficiency and thoracocentesis with diagnosis of chylothorax. During admission, a large cervico-thoracic tumor compatible with neuroblastoma was detected. The tumor included the left common carotid artery and left internal jugular vein without infiltrating them. Percutaneous biopsy revealed the diagnosis of low-risk undifferentiated neuroblastoma (group D) of the INRG classification (stage L2, age less than 18 months, MYCN not amplified, and 11q not deleted). The tumor was considered unresectable (L2), and given the symptomatology of the patient, chemotherapy treatment was initiated with significant reduction in tumor volume. After that, expectant attitude was decided upon, while the patient remained asymptomatic. In the last year, a progressive increase in tumor size was observed, so a new biopsy was performed with a diagnosis of ganglioneuroma, and surgical intervention was considered. Fig. 7 shows the comparison between MRI and virtual reconstruction. It is seen how the trachea, the internal jugular vein and the carotid artery arte desplaced by the tumor, and how the vertebral artery is encased whithin it. The vertebral artery is encompassed by the tumor. Currently, the printed models are being prepared, and the intervention will be performed soon.

## Discussion

3D technology has potential applications in medicine among which is the creation of anatomical models to plan surgeries. In this article, we report our experience in the implementation of a new high-fidelity tool for, among other specialties, pediatric oncological surgery. The 3D virtual reconstruction and prototypes, designed from both MR and CT images in the form of DICOM files, have been useful in all four cases of complex oncological resections, not only when the surgery was being planned, but have also proved to be a useful tool during the surgical intervention. 3D virtual recreation computer software allows a 360-grade view of the tumor and the relevant adjacent structures in a clear colorimetric scale, as well as modified the consistency of the structures and removes or reintroduces them as the user desired. These kinds of software calculate precisely the volume of the tumor and the normal parenchyma as well. Real-scale printed models are able to be consulted and manipulated, to corroborate the distorted and particular anatomy and vascularization of each case. In the first case (bilateral Wilms tumor), the volumetric reconstruction was decisive at the time of the planning of the intervention, proving the existence of enough remaining renal tissue, which made nephron-sparing surgery in both kidneys posible 10 (avoiding nephrectomies, one of the goals of the surgery). In this case, surgery planning with visualization and manipulation of the 3D model to achieve the greatest preservation of nephrons was an optimal tool.

Regarding case 3, in which the celiac trunk was involved and both the liver and the portal vein are displaced, the usefulness of visualization and manipulation at the pre-surgical time and during surgery was also demonstrated. This set of technologies has been widely described in in liver surgery where precise evaluations of the remaining volume of the liver and anatomical variation are needed for the preoperative planning of safe curative hepatectomy.

In surgical planning, a better understanding of spatial relationships can be obtained by exploring the surgical site interactively in a 3D format; preoperatively, this helps to evaluate more accurately the conditions under which the operation must be performed as well as to predict and prevent possible complications. 11 12 13 14 15 In addition to 3D reconstruction, the impression, manipulation, and assembly maneuvers of each structure allow to later recognize the anatomy and “not feel lost” during the dissection of unique structures, such as those found in complex solid tumors.

There are already numerous publications and surgical series in which virtual reconstruction and 3D printing prior to surgery are routinely used in multiple specialties (tumors, placement of endovascular, endobronchial prostheses, etc.). 16 17 18 19 20 New challenges are also created, such as the possibility of producing several models in series, the plasticity of the material, and its operability, etc. To summarized, it is a field open to surgery of the future, in which you can manipulate a tumor model so similar to reality that surgery could be performed in the printed model prior to the real surgery, reducing the surgical times and improving safety significantly

As an added value, 3D models the patient and his family's understanding of the intervention that will be performed, improving doctor–patient communication. 20

Our experience has been highly positive, and we believe that the application of both virtual reconstruction technology and the printing of physical models in pediatric oncological cases where complex tumor resection are required is a useful tool for improving outcome and will take a relevant place in the near future for planning and surgical training in this type of complex surgeries.

## Notes

New Insights and the Importance for a Pediatric Surgeon 3D virtual reconstruction and 3D model printing is a developing technology that has multiple potential applications in the field of surgery regarding to planning, guiding and training surgical procedures. We report our experience using this technology in pediatric surgical oncology.
